# A Low Energy–Dense Diet in the Context of a Weight-Management Program Affects Appetite Control in Overweight and Obese Women

**DOI:** 10.1093/jn/nxy041

**Published:** 2018-05-02

**Authors:** Nicola J Buckland, Diana Camidge, Fiona Croden, Jacquelynne H Lavin, R James Stubbs, Marion M Hetherington, John E Blundell, Graham Finlayson

**Affiliations:** 1 Human Appetite Research Unit, Appetite Control and Energy Balance Group, School of Psychology, University of Leeds, Leeds, United Kingdom; 2 Department of Psychology, University of Sheffield, Sheffield, United Kingdom; 3 Nutrition and Research Department, Slimming World, Alfreton, United Kingdom

**Keywords:** energy density, satiation, satiety, appetite control, weight loss, Slimming World, UK

## Abstract

**Background:**

Low energy–dense (LED) foods reduce energy intake (EI); whether this effect is sustained over time and during weight loss is unknown.

**Objective:**

This trial examined the effects of LED compared with high energy–dense (HED) meals on appetite, EI, and control over eating in the laboratory and during a weight-management program that encourages unrestricted intake of LED foods [Slimming World, UK (SW)] compared with a self-led Standard Care program [NHS weight-loss plan (SC)].

**Methods:**

Overweight and obese women [*n* = 96; mean ± SD age: 41.03 ± 12.61 y; mean ± SD body mass index (in kg/m^2^): 34.00 ± 3.61] were recruited from the SW or SC programs. Primary outcomes included appetite, food preferences (liking and wanting for LED and HED foods), cravings, and evening meal EI (LED, HED) in response to calorie-matched LED (≤0.8 kcal/g) and HED (≥2.5 kcal/g) breakfast and lunch meals. Probe-day tests were conducted at weeks 3 and 4 and repeated at weeks 12 and 13 in a within-day crossover design. Secondary outcomes, including body weight and program experience, were measured from weeks 1 to 14 in a parallel-group design. Dietary compliance was monitored with the use of weighed food diaries at weeks 3 and 12.

**Results:**

Intention-to-treat (ITT) and completers analyses showed that the SW group lost more weight than the SC group [ITT: −5.9% (95% CI: −4.7%, –7.2%) compared with −3.5% (−2.3%, −4.8%), *P* < 0.05; completers: −6.2% (−4.8%, −7.6%) compared with 3.9% (−2.5%, −5.2%), *P* < 0.05]. The SW group reported greater control over eating and more motivation to continue the program compared with the SC group. LED meals increased sensations of fullness and reduced hunger on probe days (*P* < 0.001). Total-day EI was 1057 ± 73 kcal less (95% CI: 912, 1203 kcal; 36%) under LED compared with HED conditions (*P* < .001). Liking for LED and HED foods and wanting for HED foods were lower before lunch under LED compared with HED conditions, and liking decreased to a greater extent after the LED lunch. The SW group reported fewer cravings under LED compared with HED conditions (*P* < 0.05). On probe days, appetite and EI outcomes did not differ between weeks 3 and 12 or between the SW and SC groups.

**Conclusion:**

LED meals improve appetite control in women attempting weight loss and the effect is sustainable. Consumption of LED meals likely contributed to weight loss in the SW program. This study was registered at clinicaltrials.gov as NCT02012426.

## Introduction

According to the UK National Institute for Health and Care Excellence (NICE), more high-quality trials are needed to identify effective components of weight-management programs (WMPs) (1). One component that might facilitate weight loss is the promotion of foods that are “satiety enhancing” or increase feelings of fullness (2). As hunger is a main barrier to weight loss attempts, targeting within- and between-meal satiety might be an effective strategy to improve short-term appetite control and long-term weight loss (3).

Low energy–dense (LED) foods contain fewer calories per gram than do high energy–dense (HED) foods and tend to be higher in macronutrients that are important for satiation and satiety (4, 5). Consuming LED preloads reduces hunger sensations and subsequent meal energy intake (EI) (6–8) compared with HED preloads (8) or no preloads (6) in normal-weight, overweight, and dieting individuals (6–9). The suppressant effect of LED meals on EI relative to HED foods has also been demonstrated after consuming LED meals for 2 d in normal-weight adults (10). Whether the effects of LED meals on satiation and satiety sustain over time and translate into weight loss is unclear (11–13).

Slimming World (SW), a widely available group-based commercial weight loss program in the UK and Ireland, uses a number of evidenced-based behavior change techniques to target eating and activity behaviors. The dietary component of the program, termed “Food Optimizing,” advocates ad libitum intake of many LED foods and controlled amounts of higher energy–dense foods. The effect of this dietary approach on appetite and EI has not been quantified and it is important to understand whether and how this program component may contribute to the effectiveness of the WMP for weight loss. It is possible that after following an LED diet for several weeks, compensatory responses occur, such as increased physiological hunger (14) or hedonic motivations for HED foods, which may result in greater food intake or loss of compliance (15–17). Findings from previous research are mixed: one study indicated possible increased compensatory intake after repeated consumption of LED foods (18), but another did not (19).

SW has been shown to be broadly comparable to similar commercial programs and slightly better than noncommercial programs for weight loss (20). To date, SW has not been compared with the self-led National Health Service (NHS) program, which recommends an overall 600-kcal reduction in daily EI within the context of general dietary and physical activity advice.

The primary aims of the current trial were to recruit women recently enrolled on the LED-promoting SW program or a standard care (general calorie restriction) NHS Live Well program (SC), and to examine the following: *1)* the acute effects of LED meals on subjective sensations of appetite (satiety), ad libitum EI (satiation), hedonic cravings, and preferences for HED foods; *2)* whether these acute effects diminish after a period of active weight loss; or *3)* following relatively greater consumption of LED foods in the SW program.

The secondary aims of the trial were to compare changes in body weight, body composition, and subjective experience of the WMPs in terms of convenience, psychological deprivation, control over eating, motivation, and ease of use (2).

## Method

Female participants who were overweight or obese (*n* = 96) were recruited from local Slimming World groups (SW) and the local community (SC) between September 2014 and September 2015. Recruitment emails and SW-group consultants were used to recruit newly enrolled members. The SC participants were recruited through the use of email distribution lists, University of Leeds recruitment databases (staff, students, and the community), posters, online classified adverts and social network sites. The SC participants were recruited to match the SW group in terms of age, BMI, and willingness to engage in weight loss (assessed at screening). Inclusion criteria were: female sex, aged 18–65 y, BMI (in kg/m^2^) 28–45, interested in losing weight, able to eat the study foods, and no increased physical activity in the past 4 wk. Exclusion criteria were: confounding health problems, untreated hypothyroidism, receiving systemic treatment, taking medications in the past month that have effects on appetite or weight, received bariatric surgery, pregnant (or planning), breastfeeding, known food allergies, a history of anaphylaxis to food, smokers, engaged in a commercial WMP in the preceding 2 mo, history of eating disorders, claustrophobia, worked in appetite related-research, and unable to comply with trial procedures.

On the basis of previous research (21), power calculations estimated that a power of 0.9 with a sample size of between 60 and 80 participants (30–40/group) would be sufficient to give >95% probability of detecting an effect of energy density on total within-day EI of 20–33%. This sample size was also estimated to be sufficient to detect the effect of dietary program on weight and body composition between groups.

The study conformed to relevant sections of the Declaration of Helsinki and was approved by the University of Leeds, School of Psychology ethics committee. Participants provided informed consent and upon completion received £250. The study was registered at clinicaltrials.gov as NCT02012426.

### Design

Participants were recruited to a parallel group, nonrandomized, nonblinded dietary intervention (SW, SC). The trial started with a 2-wk run-in period followed by 12 wk of trial monitoring (total 14 wk). The purpose of the run-in period was to ensure participants’ uptake and commitment to the programs (22). Participants were not blind to the WMP they were following, but were unaware that another trial group was following an alternative WMP.

To assess satiation, food preferences, and satiety outcomes, a randomized within-subjects crossover design with 2 conditions (LED, HED) was used. Participants completed 4 probe days at the Human Appetite Research Unit (HARU), where the effect of an LED diet on satiation, satiety, and food hedonics was assessed at 2 time points [weeks 3 and 4, repeated at weeks 12 and 13 (23)] (**Supplemental Figure 1**). The order of conditions was counterbalanced on the first 2 probe days and reversed for the last 2 probe days. Two time points were used to examine the sustainability of effects. On probe days, participants consumed a fixed breakfast and lunch meal and EI was assessed from the evening ad libitum meal and snacks. Participants were unaware about the nature and purpose of the energy density manipulation.

Body weight was assessed at weeks 1 and 14 and body composition and health markers were assessed after the run-in period and at week 14 (measurement sessions). Weekly questionnaires assessed WMP experience: convenience, psychological deprivation of food, motivation, satisfaction, and control over eating. Questionnaires were completed outside the research unit with instructions to complete on the same day and time each week.

### Weight-Management Programs and Dietary Advice

SW, UK is a group-based approach that involves weekly meetings as described in detail elsewhere (24). A central component of SW is a dietary approach that advocates ad libitum intake of LED foods within a balanced diet. SW consists of weekly weigh-ins, group support sessions, setting individual weight-loss goals, and access to online support (24). The SW group were asked to follow the dietary advice and full program provided by SW [for details, see (25)].

Participants in the SC group were provided with a copy of the NHS Live Well weight-loss program (26). This free, structured self-led program recommends a daily reduction of 600 kcal [NICE guidelines, (27)], provides weekly dietary and physical activity advice, and encourages recording daily EI and physical activity and weekly body weight and waist circumference (WC). Online support is also available. In terms of dietary advice, the SC group was asked to follow the diet advice from the weekly modules provided in the NHS Live Well program [for details, see (26)]. To minimize any interference with the WMPs, researchers referred participants to program resources when asked for advice.

### Study Measures

#### Subjective appetite sensations

Subjective sensations of hunger, fullness, desire to eat, and prospective consumption (PC) were collected with the use of 100-mm visual analogue scales (VASs) delivered through a portable device (28) [for specific questions and responses, see (23)]. Ratings were obtained immediately before and after every meal, before and after the Leeds Food Preference Questionnaire (LFPQ), and at hourly morning and afternoon intervals. VASs have been shown to be a valid and reliable method to assess subjective appetite sensations (29).

#### Assessment of EI

##### Test meals

The LED breakfast, lunch, evening meal, and evening snacks were designed to be ≤0.8 kcal/g based on select recipes provided by SW. Comparable HED versions were designed that were ≥2.5 kcal/g. Breakfasts and lunches contained a fixed amount of energy. Based on estimates of daily energy needs [assumed to be 1.4 × measured resting metabolic rate (RMR)] participants were allocated to 1 of 3 bands, i.e., small, ≤2000–2500 kcal/d; medium, 2501–3000 kcal/d; and large, ≥3001 kcal/d, and received a corresponding portion size. As such, this method allowed for graded individual EIs. As the participants were attempting weight loss, the total energy provided allowed for a 20% (∼600-kcal)/d energy deficit. This provided energy was distributed across the day as follows: breakfast, 20%; lunch, 30%; evening meal, ∼30%; and snacks, ∼20%. All meals were prepared in the HARU following standard operating procedures (foods sourced from Sainsbury's Supermarkets Ltd.) except for the LED evening meal (chilli con carne) which was a SW recipe, batch prepared and supplied by SW.

##### Breakfast and lunch fixed meals

The LED and HED breakfast and lunch meals provided a fixed isocaloric portion that participants were required to eat to entirety. For breakfast, the LED and HED meals consisted of a cooked breakfast with a sweet side dish. Breakfast was served with an optional tea or coffee (175 g water, plus optional 40 g semiskimmed milk). The lunches were a baked potato meal with salad and a sweet side dish (see **Supplemental Table 1** for individual food items). The LED breakfasts were lower in energy density and percentage of energy from fat and higher in weight (grams), percentage of energy from protein and carbohydrates, and grams of fiber (**Supplemental Table 2**).

##### Ad libitum evening meal and evening snacks

The LED and HED evening meals were a beef-based chilli with sides and a sweet dish (see Supplemental Table 1). The LED meal was lower in energy density, percentage of energy from fat, and grams of fiber, and was higher in percentage of energy from protein and from carbohydrates (Supplemental Table 2). Sweet and savory snacks were provided in transparent containers for evening consumption outside the laboratory (Supplemental Table 1). LED snacks were lower in energy density, percentage of energy from fat, and grams of fiber, and were higher in percentage of energy from protein and from carbohydrates (Supplemental Table 2). Following each meal, participants rated meal palatability based on appeal, pleasantness, and satisfaction with the use of 100-mm VAS.

Total daily EI (TDEI) and weight intake were determined by summing meals and snack intake.

##### Food preferences

The LFPQ (30) was used pre- and post-lunch to measure implicit and explicit food preferences for HED and LED foods. A full overview of the LFPQ method can be found elsewhere (30). In short, the LFPQ provides measures of different components of food preference and food reward. An array of food pictures were used that were either LED or HED foods but similar in familiarity, protein content, sweet or nonsweet taste, and palatability. Responses were recorded and used to compute mean scores for high-energy density, low-energy density, sweet, or savory food types (and different fat-taste combinations). To measure food liking participants rated the extent to which they liked each food (“How pleasant would it be to taste this food now?”). Food wanting was assessed with the use of a forced-choice methodology in which the food images were paired so that every image from each of the 4 food types (LED/HED, sweet/savory) is compared with every other type over repeated trials (food pairs). Participants were instructed to respond as quickly and accurately as possible to indicate the food they wanted to eat the most at that time (“Which food do you most want to eat now?”). Reaction times for all responses were covertly recorded and used to compute mean response times for each food type after adjusting for frequency of selection.

##### Cravings

At the end of each probe day, participants completed VAS that assessed craving frequency (“How often have you experienced food cravings today?”) and intensity (“How strong have any food cravings been today?”). A food craving was defined to participants as an intense desire to consume a particular food or drink that is difficult to resist.

##### Changes in body weight, body composition, RMR, and health outcomes

*Body weight, week 1.* Body weight was measured on the first day of the WMP with the use of electronic scales (SW group: recorded as part of their first weigh-in at an SW group meeting; SC group body weight was recorded by a researcher at the HARU). Weight was measured with shoes and heavy clothing removed. Height was measured at the HARU with shoes removed.

*Body composition, waist and hip circumference, weeks 2 and 14.* Fat mass (kilograms), fat free mass (kilograms), and percentage of body fat were measured by air displacement plethysmography (Bodpod, Concord) (31, 32). A researcher measured WC and hip circumference (HC) (centimeters) with a flexible nonmetal tape measure (when possible, the same researcher measured WC and HC at weeks 1 and 14). WC was measured (to the nearest 0.1 cm) at the participants’ navel at the end of an exhalation. HC was measured at the participants’ widest circumference (to the nearest 0.1 cm).

*RMR.* RMR was measured with the use of an indirect calorimeter fitted with a ventilated hood (GEM; Nutren Technology Ltd.) (33) with participants awake and lying supine for 40 min. RMR was calculated from respiratory exchange data according to the “modified” Weir equation (34). RMR was used to determine the meal bands provided on probe days.

*Resting blood pressure, heart rate, and fasting blood glucose.* Systolic and diastolic blood pressure and resting heart rate were measured in the supine position following 40 min of rest with the use of an Omron M10-IT digital blood pressure cuff.

A finger-prick blood sample was collected from participants and assessed with the use of a YSI 2300 STAT PLUS Glucose and Lactate Analyzer. For WC and HC, systolic and diastolic blood pressure, heart rate, and fasting blood glucose, 2 measures were recorded and the average was used.

*Subjective experiences of the WMPs.* Participants rated the program through the use VAS for satisfaction, contentedness, convenience, ease of use, ability to adhere to food choices, feeling in control over eating, motivation to continue, enjoyment losing weight, flexibility, feeling deprived of favorite foods, and urges to discontinue the program (“How satisfied are you with your program?”; “How convenient do you find your program?”; “How easy do you find it to stick to your program?”; “Have you felt able to stick to your plan regarding your food choices?”; “How much do you feel in control of what you're eating?”; “How motivated are you to continue with your program?”; “How enjoyable do you find losing weight with your program?”; “How flexible do you find the program”; “Generally, how deprived of your favorite foods have you felt?”; and “Have you felt the urge to rebel and abandon your program?”).

*Diet composition.* To check dietary compliance, participants completed a 7-d weighed food diary at weeks 3 and 12. Electronic scales and training were provided to ensure detailed descriptions, and consumed weights of foods and beverages were reported. Energy density was calculated from the contribution of all food and milks (excluded all other drinks) (total EI divided by total weight intake) based on criteria previously used (35).

### Procedure

#### Measurement sessions

Participants were instructed to fast from 2200 the night before, avoid alcohol the day before, and maintain similar levels of physical activity prior to sessions. Compliance was checked upon arrival. Body composition, RMR, blood pressure, fasting blood glucose, and psychometric traits (36) were then measured.

#### Probe days

Standardized control instructions were provided and compliance was checked upon arrival at the HARU. All meals and snacks were weighed pre- and post-consumption (to the nearest 0.1 g) to determine weight of food consumed. Food consumption was converted to EI according to the values provided from UK food composition tables (37) and manufacturers’ nutritional information.

### Strategy for Data Analysis

All appetite and weight outcome data were analyzed with the use of SPSS version 24 for Windows (IBM Corp). WISP 4.0 (Tinuviel Software 2013) was used to analyze food diary data. A chi-square test was conducted to compare attrition rates across groups. To compare differences between groups at baseline, a series of independent *t* tests were conducted. For primary outcomes (appetite, EI, food preferences, and cravings), mixed ANOVAs were conducted with group (SW, SC) entered as a between-subjects factor. For meal palatability values, mixed ANOVAs were conducted on appeal, pleasantness, and satisfaction scores, with condition and week as repeated-measures factors and group as a between-subjects factor. Significant interactions were explored with *t* tests based on condition averages for weeks 3 and 12. For secondary outcomes (weight, body composition, and health markers), analyses were conducted on participants who completed the trial (completers analyses) and on an intention-to-treat (ITT) basis with the use of last observation carried forward (LOCF) for missing data (38) (carrying a baseline observation forward resulted in the same outcomes for weight change though with smaller effects). Mixed ANOVAs were conducted to examine main effects and interactions between week and group on body weight and body composition. ANCOVAs were conducted on percentage of weight change controlling for starting body weight. For WMP experience, weeks 3–12 were selected and compared across week and group through the use of mixed ANOVAs. In all analyses, unless stated, condition × group and week × group interactions are reported for comparisons between groups across conditions and weeks.

All significance values *P* < 0.05 are reported. Significant interactions were explored with post-hoc analyses based on *t* tests unless stated, and a more conservative α level was set to control for multiple tests (adjusted based on the number of post-hoc comparisons; for brevity, significant interactions with nonsignificant post-hoc tests are not reported). Data are presented as means ± SEs (95% CI: lower, upper) unless specified. Partial eta squared (*η*^2^) is reported for effect sizes and interpreted as: small, 0.01; medium, 0.06; large, 0.14 (39).

## Results

### Participants

**FIGURE 1:**
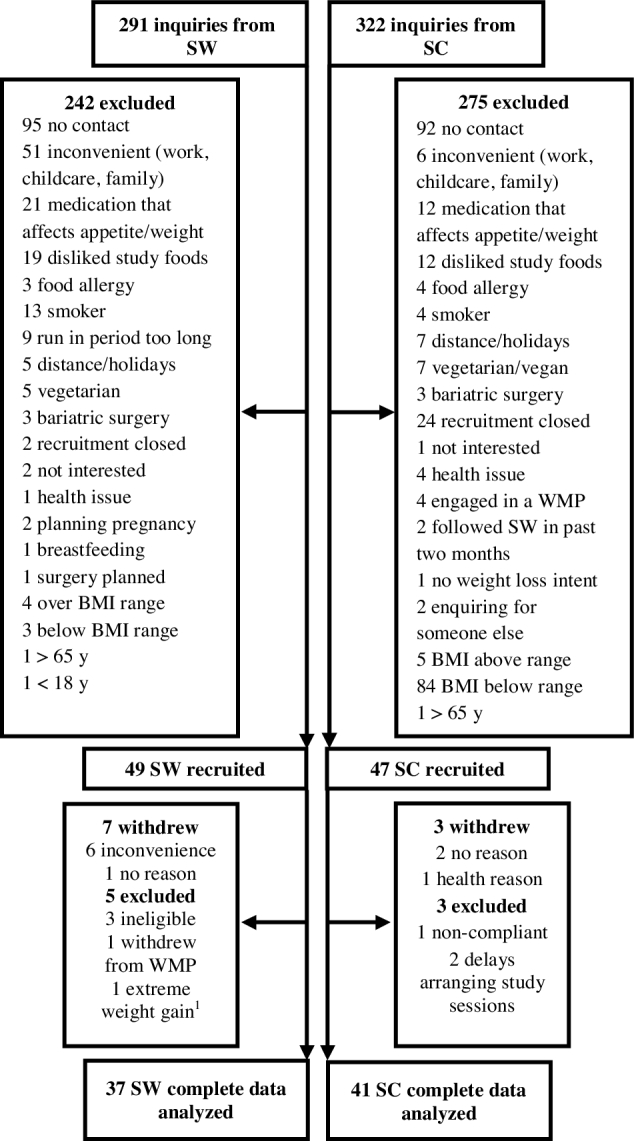
Process of recruitment and reasons for attrition and exclusions. ^1^This outlier was retained for primary analyses (e.g., appetite and energy intake) and removed from secondary analyses (e.g., weight and body composition changes). SC, Standard Care; SW, Slimming World; WMP, weight-management program.

In total, 96 participants were recruited (SW *n* = 49) (**Figure 1**). Attrition rates were similar across groups (*P* > 0.05). There were no differences in baseline measures between completers and those who withdrew or were excluded from the study (*P* > 0.05).

At weeks 1 and 2, the SW and SC groups did not differ in age, BMI, body weight, fat mass, fat-free mass, WC, HC, resting blood pressure, or fasting blood glucose (**Table 1**).

**TABLE 1:** Baseline sample characteristics for SW and SC groups^1^

	SW	SC
Age, y	41.2 ± 12.4 (37.3, 44.9)	40.9 ± 12.9 (37.0, 44.8)
Height, m	1.6 ± 0.1 (1.6, 1.7)	1.6 ± 0.1 (1.6, 1.7)
Week 1 weight, kg	92.92 ± 12.02 (89.35, 96.49)	90.57 ± 12.54 (86.89, 94.26)
Week 1 BMI, kg/m²	34.1 ± 3.7 (33.0, 35.2)	33.9 ± 3.6 (32.8, 35.0)
Week 2 weight, kg	89.99 ± 11.90 (86.37, 93.61)	88.32 ± 12.57 (84.54, 92.10)
Week 2 BMI, kg/m²	33.1 ± 3.6 (31.7, 34.2)	33.2 ± 3.6 (32.3, 34.8)
Fat mass, kg	41.75 ± 9.21 (38.67, 44.82)	40.94 ± 10.14 (37.60, 44.27)
Body fat, %	46.24 ± 5.35 (44.46, 48.02)	45.27 ± 6.33 (43.19, 47.35)
Fat free mass, kg	47.86 ± 5.86 (45.91, 49.81)	48.62 ± 6.49 (46.49, 50.75)
Waist, cm	109.8 ± 11.5 (106.3, 113.3)	107.7 ± 11.8 (104.2, 111.3)
Hip, cm	118.1 ± 9.8 (115.1, 121.1)	116.7 ± 9.5 (113.8, 119.5)
RMR, kcal/d	1674 ± 234 (1603, 1745)	1669 ± 260 (1591, 1747)
Systolic blood pressure, mm Hg	117.3 ± 14.5 (112.8, 121.7)	116.3 ± 14.9 (111.8, 120.8)
Diastolic blood pressure, mm Hg	80.4 ± 10.8 (77.1, 83.7)	79.0 ± 9.7 (76.1, 81.9)
Heart rate, bpm	63.3 ± 8.7 (60.7, 66.0)	61.8 ± 8.3 (59.3, 64.4)
Blood glucose, mmol/L	4.9 ± 0.7 (4.7, 5.1)	4.8 ± 0.7 (4.6, 5.1)
Cognitive restraint^2^	10 ± 4 (9, 11)	9 ± 4 (8, 10)
Disinhibition^2^	10 ± 3 (9, 11)	11 ± 3 (10, 12)
Hunger^2^	6 ± 4 (5, 8)	7 ± 3 (6, 8)

^1^Values are means ± SDs (95% CI). SW, *n* = 44; SC, *n* = 45. RMR, resting metabolic rate; SC, standard care; SW, Slimming World.

^2^Cognitive restraint, disinhibition, and hunger scores were assessed at week 2 with the Three Factor Eating Questionnaire (36).

### Subjective sensations of appetite

Hunger, desire to eat, PC, and fullness profiles are shown in **Supplemental Figure 2**. Hunger, desire to eat, and PC were lower at every time point on the LED days than on the HED days [*ŋp*² = 0.22, *P* < 0.001] with the exception of prebreakfast and postevening meal, whereas fullness was significantly higher at every time point throughout the day with the exception of pre-breakfast [*ŋp*² = 0.22; *P* < 0.001]. Responses did not differ between groups or weeks (all *P* > 0.05).

### Ad libitum evening meal, ad libitum snack intake, and total-day EI (kilocalories and grams)

Compared with the HED days, on LED days participants consumed significantly less energy from the evening ad libitum meal [LED: 611 ± 25 kcal (562, 661 kcal); HED: 1219 ± 50 kcal (1112, 1318 kcal), *ŋp*² = 0.79; *P* < 0.001] and snacks [LED: 307 ± 18 kcal (271, 343 kcal); HED: 757 ± 64 kcal (629, 885 kcal), *ŋp*² = 0.47; *P* < 0.001]. This resulted in a significant TDEI reduction of 1057 ± 73 kcal (912, 1203 kcal) (36%) under LED conditions compared with HED conditions [mean TDEI: LED: 1901 ± 38 kcal (1826, 1975 kcal); HED: 2958 ± 97 kcal (2764, 3152 kcal), *ŋp*² = 0.74; *P* < 0.001].

For weight consumed, participants consumed 236 ± 21 g more of the LED evening meal compared with the HED evening meal [LED: 753 ± 31 g (692, 813 g); HED: 517 ± 20 g (478, 556 g), *ŋp*² = 0.62; *P* < 0.001]. There were no significant differences between conditions in snack weight intake [LED: 225 ± 17 g (191, 260 g); HED: 206 ± 17 g (171, 240 g), *ŋp*² = 0.01; *P* > 0.05]. Total-day weight intake was 1212 ± 29 g greater under LED conditions than under HED conditions [LED: 2301 ± 43 g (2216, 2386 g); HED: 1089 ± 30 g (1029, 1149 g), *ŋp*² = 0.96; *P* < 0.001]. EI and weight intake did not differ across groups or weeks [*ŋp*² = 0.02; *P* > 0.05].

Despite consuming less energy from the LED evening meal, participants rated the LED evening meal as more appealing, pleasant, and satisfying (*P* < 0.03) than the HED evening meal (**Supplemental Table 3**).

### Food preferences: explicit liking and implicit wanting for LED and HED foods

Prelunch, liking for all foods was lower under LED conditions than under HED conditions (*ŋp*² = 0.28, *P* < 0.001). Across both conditions, from pre- to postlunch, liking decreased for all foods (*ŋp*² = 0.77; *P <* 0.001). However, liking for all foods reduced to a greater degree after the LED lunch compared with the HED lunch [LED: −28.49; HED; −17.34, *ŋp*² = 0.28; *P* < 0.001]. This reduction in liking following the LED lunch was specifically greater for HED foods than for LED foods (significant condition × time × energy density interaction, *ŋp*² = 0.11, *P* < 0.01). Overall liking for food did not differ between groups or between weeks (*P* > 0.05).

In the LED condition, mean wanting for HED foods was lower at prelunch compared with the HED condition (significant diet × time × food type interaction on wanting, *ŋp*² = 0.08, *P* < 0.05). In the LED condition, wanting for HED foods was significantly lower in the SW (−3.81 ± 2.89 RT/freq) than in the SC group (0.97 ± 3.01 RT/freq) as qualified with a significant group × condition × food type interaction on wanting for high-fat foods, *ŋp*² = 0.11, *P* < 0.01. Wanting for HED foods in the HED condition did not differ between groups or weeks (*P* > 0.05).

### Cravings

For the SC group, craving frequency and intensity did not significantly differ between the LED and HED probe days. For the SW group, however, craving frequency and intensity were lower on the LED probe days than on the HED probe days [frequency: SC: LED: 23.3 ± 4.2 mm (14.9, 31.8 mm); HED: 31.3 ± 4.3 mm (22.6, 39.9 mm); SW: LED: 17.8 ± 3.2 mm (11.4, 24.3 mm); HED: 43.3 ± 4.6 mm (33.9, 52.8 mm); intensity: SC: LED: 24.7 ± 3.6 mm (17.6, 31.8 mm); HED: 34.1 ± 4.4 mm (25.3, 42.9 mm); SW: LED: 19.3 ± 3.8 mm (11.7, 26.8 mm); HED: 41.9 ± 4.7 mm (32.6, 51.2 mm)*, ŋp*² = 0.06; *P* < 0.05). Craving frequency and intensity did not differ across weeks (*ŋp*² < 0.01; *P* > 0.05).

### Effects of WMP on changes in weight, body composition, and health markers

#### Changes in weight between week 1 and week 14

**Table 2** shows mean changes in weight, body composition, and health markers for LOCF and completers analyses. Weight loss was significant for both groups (*P* < 0.001). The SW group lost more weight than the SC-group [the loss remained significant when controlling for baseline body weight (*P* < 0.05)]. The SW were more likely to lose clinically significant amounts of weight loss (>5%) than the SC group [χ: χ(1) = 6.69, *P* < 0.05] (**Supplemental Figure 3**).

**TABLE 2:** Changes in body weight, body composition, and health markers in SW and SC groups after a 14-wk program^1^

	*n*	SW	SC
% Weight change^2^			
LOCF	92	−5.94 (−4.66, −7.23)	−3.52 (−2.26, −4.78)*
Completers	78	−6.21 (−4.81, −7.62)	−3.85 (−2.51, −5.18)*
Body weight,^2^ kg			
LOCF	92	−5.51 (−4.36, −6.66)	−3.06 (−1.93, −4.18)**
Completers	78	−5.81 (−4.55, −7.01)	−3.32 (−2.13, −4.51)**
Fat mass,^2^ kg			
LOCF	75	−2.25 (−1.32, −3.18)	−1.06 (−0.15, −1.98)
Completers	65	−2.90 (−1.91, −3.89)	−1.06 (−0.12, −2.01)*
% Fat^2^			
LOCF	75	−1.54 (−0.84, −2.25)	−0.66 (0.04, −1.35)
Completers	65	−1.98 (−1.22, −2.74)	−0.67 (0.06, −1.40)*
Fat free mass,^2^ kg			
LOCF	75	0.14 (0.49, −0.21)	−0.15 (0.20, −0.50)
Completers	65	0.17 (0.55, −0.21)	−0.15 (0.21, −0.51)
Waist circumference, cm			
LOCF	88	−2.7 (−1.3, −4.1)	−2.6 (−1.2, −4.0)
Completers	75	−3.2 (−1.6, −4.7)	−2.0 (−0.7, −3.2)
Hip circumference, cm			
LOCF	88	−1.8 (−0.3, −3.3)	−1.3 (−0.1, −2.5)
Completers	78	−2.0 (−0.3, −3.6)	−1.4 (−0.1, −2.6)
Resting metabolic rate, kcal/d			
LOCF	80	−127 (−80, −173)	12 (85, −62)**
Completers	70	−148 (−101, −196)	10 (89, −70)**
Fasting blood glucose, mmol			
LOCF	84	−0.25 (−0.03, −0.47)	−0.27 (−0.03, −0.51)
Completers	74	−0.28 (−0.02, −0.53)	−0.24 (0.02, −0.50)
Systolic blood pressure, mm Hg			
LOCF	87	1.1 (−2.6, 4.8)	0.8 (−2.2, 3.9)
Completers	77	0.7 (−3.5, 4.9)	0.0 (−3.0, 3.0)
Diastolic blood pressure, mm Hg			
LOCF	87	−0.0 (−2.5, 2.5)	0.4 (−2.1, 3.0)
Completers	77	0.1 (−2.8, 3.1)	0.2 (−2.4, 2.9)
Resting heart rate, bpm			
LOCF	86	0.5 (−2.1, 3.1)	0.02 (−1.6, 1.6)
Completers	76	0.8 (−2.1, 3.8)	−0.01 (−1.8, 1.7)

^1^Values are mean changes between week 1 (percentage of weight change and body weight) or 2 (all other measures) and week 14 (95% CIs). LOCF and completers anaylsis presented. *,**Different from SW, *P < 0.05; **P < 0.01. LOCF, last observation carried forward; SC, standard care; SW, Slimming World.

^2^Analyses controlled for method of assessing body composition (BodPod or bioelectrical impedance).

#### Changes in body composition

For changes in body composition, data were missing for 13 completers (SW: *n* = 6) because a technical failure meant the BodPod could not be used at both time points to measure body composition. The body composition of a further 4 participants was measured at both time points by bioelectrical impedance (model BC418MA, Tanita) and due to consistency these data were included in the analyses (included as a covariate in the analysis). As such, data reported for completers was for a sample size of 65 (SW: *n* = 31). The SW group lost more fat mass and greater percentage of fat than the SC group (LOCF analyses were not significant) and this remained significant when controlling for starting fat mass (*P* < 0.05). Fat-free mass did not change between weeks for either groups. WC and HC reduced in both groups (*P* < 0.01), though this reduction did not differ between groups.

#### Changes in RMR and health markers

RMR significantly decreased in the SW group but did not change in the SC group. Fasting blood glucose decreased in both groups (*P* = 0.005), this reduction did not differ between groups. Systolic and diastolic blood pressure and resting heart rate did not change in either group (Table 2).

#### Experiences of the WMP

The average response rate to weekly questionnaires was 84.8% ± 27.2%. The SW group felt the WMP was easier to stick to, felt more in control over their eating and food choices, experienced more enjoyment as they lost weight, were more satisfied, and were more motivated to continue the program compared with the SC group (Table 3). There were no differences between groups on rated convenience, urges to rebel and abandon the WMP, flexibility, or feeling deprived of favorite foods (Table 3).

**TABLE 3:** Weekly experiences of the weight-management program reported by SW and SC groups^1^

VAS item, mm	SW	SC
Satisfaction	72.8 (64.5, 81.0)	54.2 (46.0, 62.3)**
Content	69.8 (61.4, 78.1)	50.1 (42.0, 58.3)**
Convenient	71.5 (64.1, 78.9)	62.7 (55.6, 69.9)
Easy to stick to	64.5 (56.8, 72.2)	45.5 (37.9, 53.1)**
Adhere to food choices	59.5 (52.2, 66.8)	44.5 (37.4, 51.6)**
In control	69.0 (61.0, 77.1)	54.3 (46.4, 62.2)*
Motivated	76.2 (68.2, 84.2)	60.1 (52.2, 68.0)**
Enjoyment	71.9 (63.0, 80.9)	53.0 (44.2, 61.8)**
Flexibility	70.8 (63.5, 78.1)	67.6 (60.4, 74.7)
Deprived	30.8 (24.0, 37.6)	30.4 (23.7, 37.1)
Abandon program	39.0 (30.7, 47.3)	43.8 (35.3, 52.3)

^1^Values are means (95% CIs) based on SW *n* = 26; SC *n* = 27. Completers analysis shown. Results were the same for intention-to-treat analyses (with the use of last observation carried forward). *,**Different from SW, *P < 0.05, **P < 0.01. SC, standard care; SW, Slimming World; VAS, visual analogue scale.

#### Diet composition

The food diaries showed that at both time points (weeks 3 and 12) the SW group's diet was less energy–dense than the SC group's diet [week 3: SW: 1.14 kcal/g (4.78 kJ/g), SC: 1.51 kcal/g (6.33 kJ/g); week 12: SW: 1.27 kcal/g (5.33 kJ/g), SC: 1.56 kcal/g (6.54 kJ/g), *p* < 0.001].

## Discussion

The current findings demonstrate the utility of LED meals for reducing subjective sensations of appetite and meal EI in overweight or obese women during active weight loss. The effects of LED meals were sustained following a 14-wk WMP, which included either a dietary component that promotes ad libitum intake of LED foods (Slimming World, UK), or a standard care WMP based on national guidelines for weight loss. The SW program was associated with greater reductions in weight and fat mass, and greater ease, enjoyment, satisfaction, and motivation to continue with the program compared with SC. Despite differences in weight outcomes, there were no differential effects of the WMPs on appetite sensations or EI in response to LED compared with HED foods as measured in the laboratory. Both WMPs resulted in reduced fasting blood glucose and no increases in blood pressure.

The LED meals increased sensations of fullness and reduced hunger, desire to eat, and PC throughout the day. This resulted in lower TDEI. These findings correspond with previous research which reported reduced EI in normal-weight women after consuming LED meals for 2 d (10) and extend these findings to women who were engaged in a weight-loss program. Reduced EI occurred without increasing hedonic wanting or subjective cravings. This novel finding is important because energy-reducing diets can lead to increases in the reinforcing value of food (40) and the appeal of high-fat foods (41). As such, an LED diet appears to be effective in reducing EI while limiting hedonic motivations and promoting dietary control, at least over the time window of the study.

The findings suggest that the effects of LED meals on satiation and satiety were sustained after following an LED diet for 12 wk (based on probe-day assessments at week 12). Thus, adherence to an LED diet offers a potentially effective strategy to assist weight loss by promoting satiation and satiety. Adherence to an LED diet may help limit the effect of physiologic changes that weaken satiety and promote weight regain subsequent to weight loss (42). Indeed, previous research has reported an association between LED diets and lower increases in the hunger hormone ghrelin in response to weight loss (43). Future research should examine gut peptide responses to acute and more prolonged consumption of an LED diet to understand the potential for physiological responses to lead to compensation or loss of compliance in the long term.

Despite lower EIs, weighed intake of the ad libitum LED meal was higher compared with HED conditions. LED foods may exert a constraining effect on caloric compensation (44, 45). LED foods may also induce greater satiation and satiety due to increased oral processing times (46), altered gut hormones (47), cognitive factors (8), and higher intake of protein and fiber (4, 5). It is important to note that in this study, the LED and HED meals varied not only in the energy density but also in weight, fat, protein, carbohydrates, and grams of fiber. It is likely that the effects observed are due to a combination of these varying nutritional properties of LED meals and not energy density alone. This study used a whole-diet approach rather than isolating a specific nutrient, and this allowed us to use meals that reflect those consumed by individuals engaged in weight loss.

These findings support previous research documenting the effectiveness of commercial behavior change programs for weight loss (24, 48–58). Most of this evidence is from 12-wk free primary care partnership schemes (20, 48, 49, 53, 54). Similar results have been reported in regular fee-paying programs (24, 50). The current study extends these findings by demonstrating the effectiveness of SW for weight loss and changes in body composition compared with the SC program in women who self-referred to a weight-loss program. The findings, along with previous research, suggest that evidence-based structured programs are more effective for weight and fat loss than self-led approaches [e.g. (52)].

The current trial also examined experiential aspects of the commercial WMP. The SW group felt more in control over what they were eating and abler to comply with food choices than did the SC group, which is consistent with the appetite and eating behavior responses exhibited by the sample overall in response to the LED probe days in the laboratory. The SW group experienced greater satisfaction, enjoyment, and motivation to continue, suggesting that the SW may have been be easier for people to follow and adhere to compared with an SC approach, although it is not possible to specify which program components led to these differences in overall program evaluation.

The present study used a nonrandomized, parallel groups design, which may limit the certainty of some findings (59). However, the 2 groups were matched at baseline in terms of age, motivation to lose weight, eating behavior traits (in preparation), body composition, and health measures. Nevertheless, an issue with the nonrandomized design is that participants’ body composition, health markers, and appetite measures were not assessed before starting the program. Although this issue does not affect weight change (recorded at weeks 1 and 14), it is important to consider that initial enrolment in the program might have minimized the opportunity to observe differences between groups on these outcomes.

The trial did not address long-term outcomes and it is well established that there is a tendency for weight to be regained after 6 mo, at least in ITT models (60). The focus of the current research was to understand the mechanisms of LED meals on initial weight loss. However, given that weight loss maintenance is frequently the greater challenge (61), more trials should test the effects of LED strategies on long-term weight loss maintenance. Although not demonstrated in the time frame used in this study, it is possible that after following an LED diet for a sustained period, individuals learn to associate the sensory properties of LED meals with low energy and compensate by increasing portion size or seeking higher energy–dense foods (17, 18). Thus, it would be useful to repeat this study and compare responses to LED meals after initial enrolment and sustained engagement (e.g., 1 y) in an LED program.

Additionally, although the SW and SC groups were generically similar in the advice given, they differed considerably in the specific dietary recommendations, mode of delivery, intensity, peer-group support, and implementation strategies. This study primarily focused on the effects of specific dietary advice provided and how that may have influenced eating behavior in the laboratory. It is unlikely that the group difference in weight change was entirely ascribable to dietary factors alone. Thus, the effects should not be overestimated or extrapolated to other program components that differed (62). The present study also did not use specific behavior change taxonomies to characterize and compare how specific program characteristics may have affected weight outcomes (63). Nevertheless, despite these limitations, it remains highly plausible that within a multicomponent program, the promotion of LED meals can contribute to improved appetite control and weight management.

In summary, this study provided the first evidence that LED meals delivered in the context of weight loss reduce subjective appetite and hedonic motivations to eat, increase control over eating, and reduce TDEI compared with HED meals. These effects were sustained after prolonged engagement in 2 different WMPs. Promoting consumption of LED meals is likely to contribute to the significant weight loss and reductions in fat mass observed in women following the SW program.

## Supplementary Material

Supplemental data

## Notes

Funded by Slimming World UK. Slimming World UK supported the design of the test meals and recruitment.

Author disclosures: JHL is employed by Slimming World UK; RJS consults for Slimming World through the University of Leeds consulting service. NJB, DC, FC, MMH, JEB, and GF, no conflicts of interest.

Supplemental Tables 1–3 and Supplemental Figures 1–3 are available from the “Supplementary data” link in the online posting of the article and from the same link in the online table of contents at https://academic.oup.com/jn/.

Abbreviations used: EI, energy intake; HARU, Human Appetite Research Unit; HC, hip circumference; HED, high energy dense; ITT, intention-to-treat; LED, low energy dense; LFPQ, Leeds Food Preference Questionnaire; LOCF, last observation carried forward; NHS, National Health Service; NICE, National Institute for Health and Care Excellence; PC, prospective consumption; RMR, resting metabolic rate; SC, standard care; SW, Slimming World; TDEI, total daily energy intake; VAS, visual analogue scale; WC, waist circumference; WMP, weight-management program.
