# Supplementary material for: A Low Energy–Dense Diet in the Context of a Weight-Management Program Affects Appetite Control in Overweight and Obese Women
Source: J Nutr. 2018 May 2;148(5):798–806. doi: 10.1093/jn/nxy041 (PMC6054218; doi:10.1093/jn/nxy041)
Supplement: Supplemental data [file nxy041_supp.zip › nut264432-file001.docx]

**Supplemental Table 1.** Energy density for the food items provided to Slimming World and Standard Care groups on the LED and HED probe days

| **Food** | **Energy density (kcal/g)** | | |
| --- | --- | --- | --- |
| **LED breakfast** |  |  | |
| Toast (wholemeal) |  | 2.13 | |
| Margarine (light) |  | 2.81 | |
| Scrambled eggs |  | 1.60 | |
| Baked beans |  | 0.69 | |
| Mushrooms |  | 0.13 | |
| Tomatoes |  | 0.14 | |
| Mango |  | 0.58 | |
| Strawberries |  | 0.27 | |
| Grapes |  | 0.60 | |
| Clementine |  | 0.35 | |
| **HED breakfast** |  |  | |
| Toast (wholemeal, seeded) |  | 2.57 | |
| Butter |  | 7.23 | |
| Scrambled eggs (cream/butter) |  | 2.66 | |
| Tomatoes |  | 0.14 | |
| Mushrooms |  | 0.13 | |
| Danish pastry |  | 4.86 | |
|  | | |  |
| **LED lunch** | | |  |
| Baked potato |  | 0.93 | |
| Tuna (in water) |  | 1.13 | |
| Sweetcorn |  | 1.05 | |
| Mayonnaise |  | 7.21 | |
| Crème fraiche (light) |  | 1.62 | |
| Lettuce |  | 0.13 | |
| Tomatoes |  | 0.14 | |
| Yellow peppers |  | 0.27 | |
| Cucumber |  | 0.09 | |
| Red onions |  | 0.36 | |
| Sweet chilli dressing (light) |  | 0.41 | |
| Yogurt, strawberry (low fat). |  | 0.47 | |
| **HED lunch** |  |  | |
| Baked potato |  | 0.93 | |
| Tuna (in brine) |  | 1.89 | |
| Sunflower oil (mixed in tuna) |  | 8.28 | |
| Mayonnaise |  | 7.21 | |
| Lettuce |  | 0.13 | |
| Tomatoes |  | 0.14 | |
| Olive oil dressing |  | 8.23 | |
| Mousse, chocolate |  | 3.09 | |
|  |  |  | |
| **LED evening meal** |  |  | |
| Beef chilli |  | 0.68 | |
| Rice |  | 1.33 | |
| Cheese (light) |  | 3.23 | |
| Yellow peppers |  | 0.27 | |
| Red onion |  | 0.36 | |
| Lettuce |  | 0.13 | |
| Tomatoes |  | 0.14 | |
| **LED evening meal (continued)** |  |  | |
| Cucumber |  | 0.09 | |
| Banana |  | 0.95 | |
| Yogurt (low fat) |  | 0.44 | |
| Sweetener |  | 3.57 | |
| **HED evening meal** |  |  | |
| Beef chilli |  | 1.22 | |
| Tortilla chips |  | 4.70 | |
| Cheese |  | 3.89 | |
| Guacamole |  | 2.04 | |
| Sour cream |  | 1.86 | |
| Mayonnaise^1^ |  | 7.21 | |
| Double cream^1^ |  | 4.39 | |
| Chocolate brownies |  | 4.18 | |
|  |  |  | |
| **LED snacks** |  |  | |
| Yogurt |  | 0.47 | |
| Grapes |  | 0.60 | |
| Pineapple |  | 0.52 | |
| Carrots |  | 0.33 | |
| Cucumber |  | 0.09 | |
| Cottage cheese |  | 0.69 | |
| Ham |  | 1.18 | |
| Chocolate |  | 4.30 | |
| Crisps, ready salted |  | 4.08 | |
| **HED snacks** |  |  | |
| Yogurt (chocolate) |  | 2.49 | |
| Jelly sweets (soft) |  | 3.13 | |
| Cheese savory twists |  | 4.92 | |
| Crackerbread with seeds |  | 3.70 | |
| Hummus |  | 2.65 | |
| Sausages |  | 2.91 | |
| Chocolate |  | 5.17 | |
| Flapjacks |  | 4.15 | |
| Crisps, ready salted |  | 5.05 | |

^1^Mayonnaise and double cream were mixed into the guacamole and sour cream (50/50 proportions).

^2^Energy density was sourced between January and August 2014 from manufacturers’ product nutritional information and the UK Composition of Foods Database (1).

^3^HED, high energy density; LED, low energy density.

**Supplemental Table 2.** Serving size and nutritional information for the LED and HED fixed-caloric meals and ad libitum evening meal and snacks that were provided to Slimming World and Standard Care groups on probe days.

| **Meal** | **Serving**  **(g)** | **Energy**  **(kcal)** | **Protein**  **(g)** | **Fat**  **(g)** | **CHO**  **(g)** | **Fiber**  **(g)** | **% energy from Pro** | **% energy from Fat** | **% energy from CHO** | **ED**  **(kcal/g)** |
| --- | --- | --- | --- | --- | --- | --- | --- | --- | --- | --- |
| **Breakfast** |  |  |  |  |  |  |  |  |  |  |
| LED | 544.5 | 360.0 | 17.6 | 8.5 | 56.7 | 10.3 | 19.6 | 21.4 | 59.1 | 0.7 |
| HED | 122.0 | 360.1 | 9.5 | 26.1 | 23.4 | 2.2 | 10.5 | 65.1 | 24.4 | 3.0 |
| **Lunch** |  |  |  |  |  |  |  |  |  |  |
| LED | 693.7 | 540.0 | 37.3 | 16.9 | 63.6 | 6.5 | 27.6 | 28.2 | 44.2 | 0.8 |
| HED | 217.8 | 540.0 | 14.0 | 39.7 | 33.8 | 2.1 | 10.3 | 66.2 | 23.4 | 2.5 |
| **Evening meal** |  |  |  |  |  |  |  |  |  |  |
| LED | 2358 | 1789 | 127.4 | 26.8 | 276.9 | 23.6 | 28.5 | 13.5 | 58.1 | 0.8 |
| HED | 1839 | 4730 | 124.7 | 305.0 | 396.0 | 65.0 | 10.5 | 58.0 | 31.4 | 2.6 |
| **Snacks** |  |  |  |  |  |  |  |  |  |  |
| LED | 1073 | 909.2 | 48.3 | 19.6 | 143.8 | 9.8 | 21.3 | 19.4 | 59.3 | 0.8 |
| HED | 1073 | 3915 | 80.4 | 199.1 | 480.4 | 29.2 | 8.2 | 45.8 | 46.0 | 3.6 |

^1^The serving size provided was based on estimates of daily energy needs which determined whether participants received a small (≤2000-2500 kcal/day), medium (2501-3000 kcal/day) or large (≥3001 kcal/day) portion. The small portion is shown. The proportion of macronutrients remained the same across the three portions.

^2^ED, energy density; HED, high energy density; LED, low energy density.

**Supplemental Table 3.** Palatability scores (mm) for the LED and HED meals rated by Slimming World and Standard Care groups.

|  | **Slimming World** | | | **Standard Care** | | | **Mixed ANOVA (*p*)** | | |
| --- | --- | --- | --- | --- | --- | --- | --- | --- | --- |
|  | | **LED** | **HED** |  | **LED** | **HED** | **Group** | **Condition** | **Interaction** |
| **Appeal** | |  |  |  |  |  |  |  |  |
| Breakfast^2^ | | 86.90 ± 2.56 | 66.61 ± 3.78** |  | 78.31 ± 2.46 | 78.83 ± 3.63 | 0.57 | 0.01 | 0.01 |
| Lunch^2^ | | 88.48 ± 3.05 | 61.23 ± 3.97*** |  | 79.53 ± 2.93 | 77.92 ± 3.81 | 0.31 | <0.001 | <0.001 |
| Evening meal^3^ | | 91.38 ± 2.85 | 86.19 ± 3.22* |  | 78.65 ± 2.75 | 75.55 ± 3.10* | 0.01 | 0.03 | 0.59 |
| **Pleasant** | |  |  |  |  |  |  |  |  |
| Breakfast^2^ | | 85.69 ± 2.80 | 76.77 ± 2.94 |  | 78.75 ± 2.69 | 83.34 ± 2.82 | 0.46 | 0.95 | 0.02 |
| Lunch^4^ | | 90.03 ± 2.87 | 76.12 ± 3.13*** |  | 78.52 ± 2.80 | 82.12 ± 3.05 | 0.39 | 0.06 | 0.01 |
| Evening meal^5^ | | 92.33 ± 2.48 | 84.34 ± 3.27* |  | 81.71 ± 2.42 | 78.24 ± 3.19* | 0.02 | 0.01 | 0.24 |
| **Satisfaction** | |  |  |  |  |  |  |  |  |
| Breakfast^2^ | | 93.07 ± 2.40 | 64.00 ± 3.81*** |  | 84.75 ± 2.31 | 74.38 ± 3.66 | 0.73 | <0.001 | 0.01 |
| Lunch^4^ | | 93.96 ± 2.22 | 56.10 ± 3.43*** |  | 84.96 ± 2.16 | 74.14 ± 3.34** | 0.14 | <0.001 | <0.001 |
| Evening meal^5^ | | 94.54 ± 2.21 | 84.76 ± 3.39* |  | 85.06 ± 2.16 | 83.81 ± 3.31* | 0.11 | 0.02 | 0.07 |

^1^Values are means ± SEM.

^2^n = 36; SC n = 39

^3^SW n = 38; SC n = 41

^4^SW n = 37; SC n = 39

^5^SW n = 38; SC n = 40.

^6^*Different from LED, *p*<0.025; **Different from LED, *p*<0.01; ***Different from LED, *p*<.001.

^7^HED, high energy density; LED, low energy density.

**Supplemental references**

1 Finglas, P. M. *et al.* *McCance and Widdowson's The Composition of Foods*. Seventh summary edition, Royal Society of Chemistry, 2015.
