# Supplementary material for: A Low Energy–Dense Diet in the Context of a Weight-Management Program Affects Appetite Control in Overweight and Obese Women
Source: J Nutr. 2018 May 2;148(5):798–806. doi: 10.1093/jn/nxy041 (PMC6054218; doi:10.1093/jn/nxy041)
Supplement: Supplemental data [file nxy041_supp.zip › nut264432-file002.pdf]

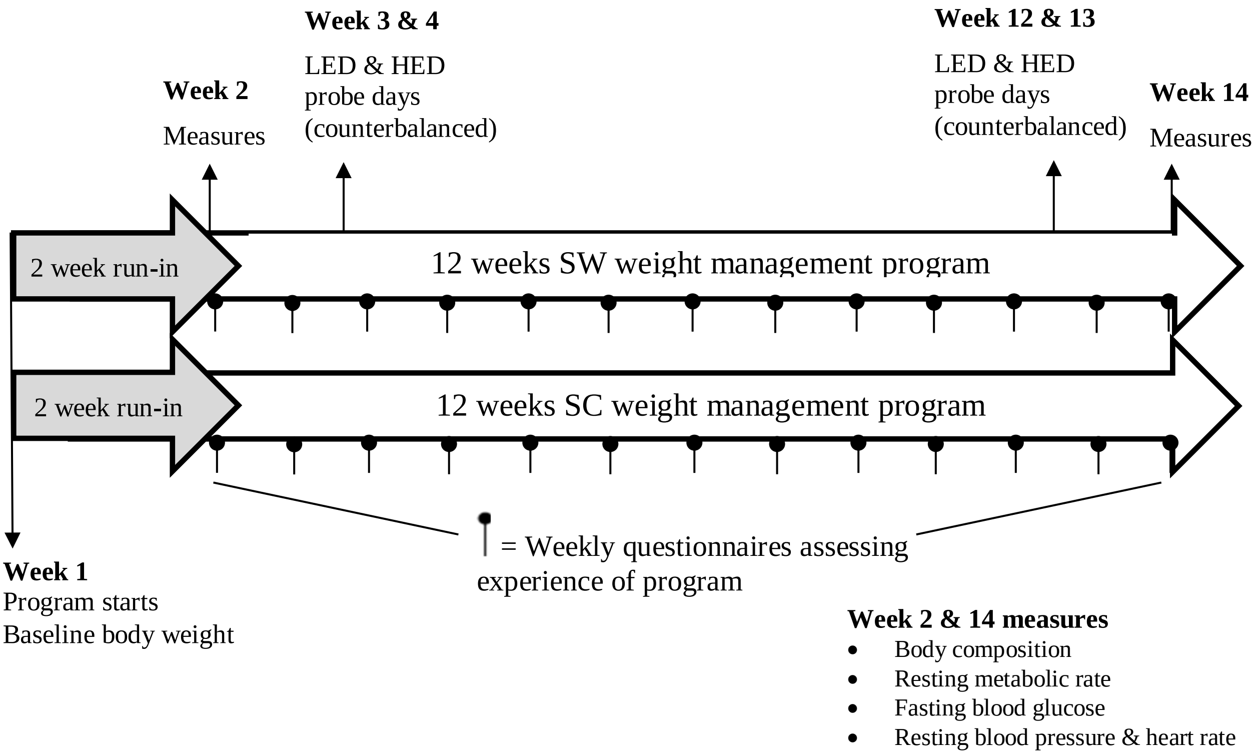


**Supplemental Figure 1.** Study design and procedure for Slimming World (SW) and Standard Care (SC) groups

[1]HED, high energy density; LED, low energy density.

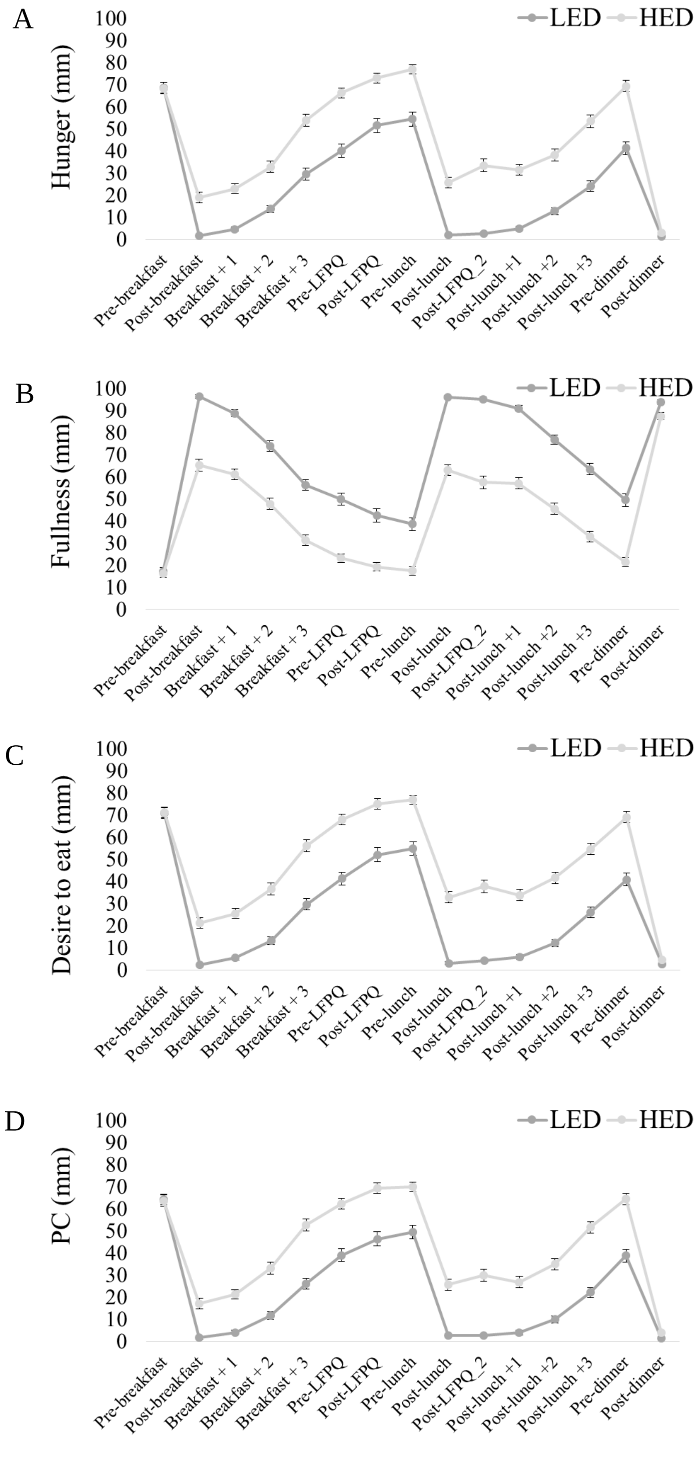


**Supplemental Figure 2.** Hunger (A), fullness (B), desire to eat (C) and prospective consumption (PC) (D) profiles on the LED and HED probe days (M ± SEM) in the Slimming World (SW) and Standard Care (SC) groups.

[1]Values are mean ± SEM based on n = 76; Data collapsed across SW and SC groups and weeks 3 and 12 because SC was not different from SW and ratings at week 12 were not different from ratings at week 3.
[2]HED, high energy density; LED, low energy density.

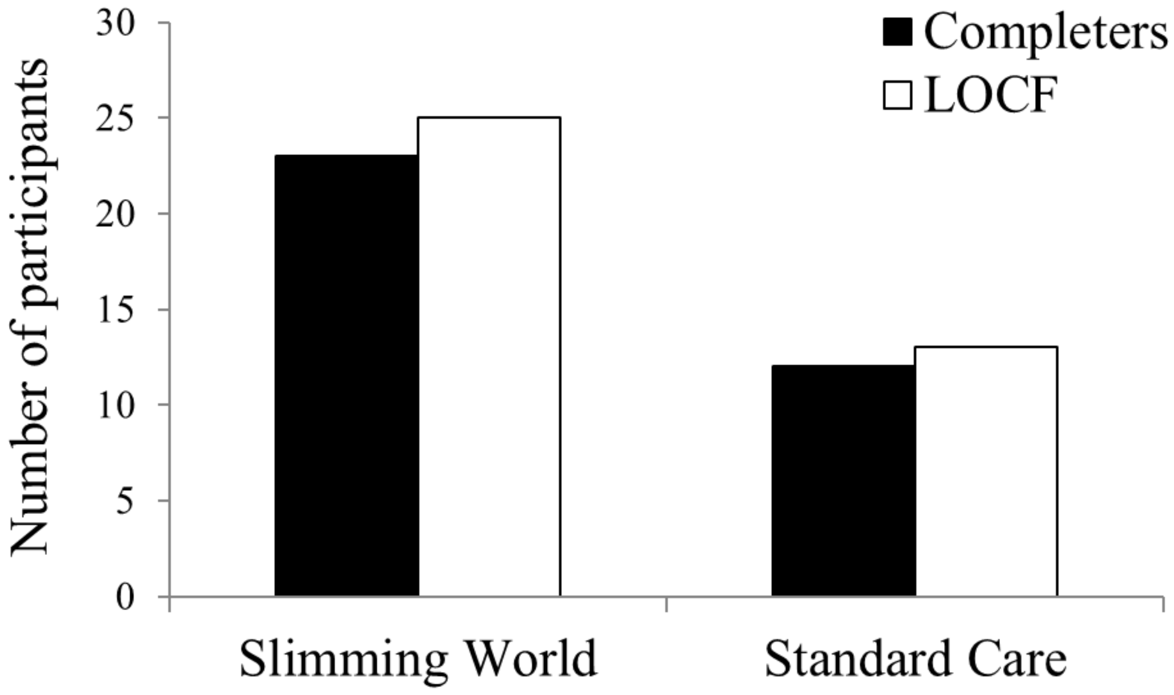


**Supplemental Figure 3**. Number of participants from the Slimming World and Standard Care groups achieving 5% weight loss between week 1 and 14 for completers and accounting for study attrition (LOCF).
[1]LOCF, last observation carried forward.
